# Supplementary material for: Ultrastructure of the axonal periodic scaffold reveals a braid-like organization of actin rings
Source: Nat Commun. 2019 Dec 20;10:5803. doi: 10.1038/s41467-019-13835-6 (PMC6925202; doi:10.1038/s41467-019-13835-6)
Supplement: Supplementary file 1 — Supplementary Information [file 41467_2019_13835_MOESM1_ESM.pdf]

Supplementary Information for:

Ultrastructure of the axonal periodic scaffold reveals a braid-like organization of actin rings

Stéphane Vassilopoulos, Solène Gibaud, Angélique Jimenez, Ghislaine Caillol, Christophe Leterrier

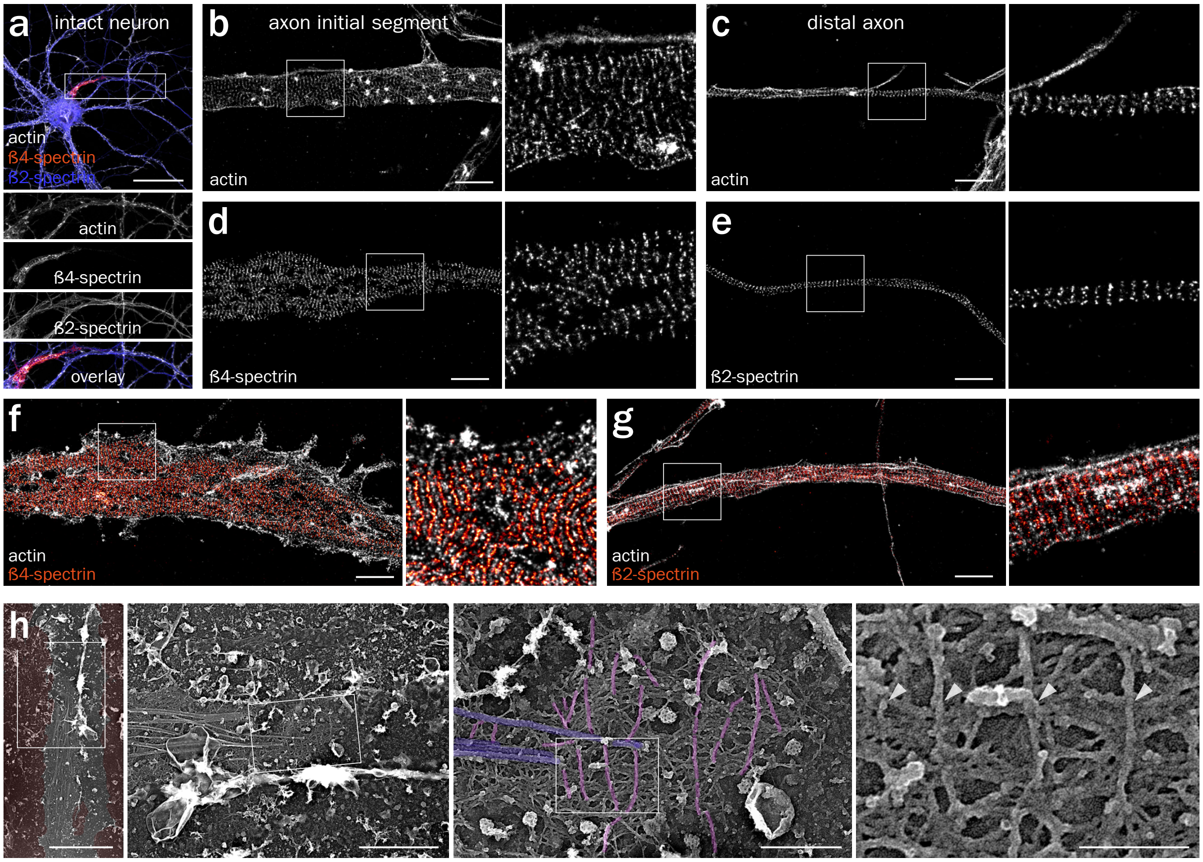

Supplementary Figure 1. SMLM of the MPS along intact axons and additional PREM views of an unroofed axon.
(a) Epifluorescence image of a neuron labeled for actin (gray), ß4-spectrin (orange) and ß2-spectrin (blue). **(b-c)** SMLM images showing actin rings along the AIS (b) and the distal axon (c). **(d-e)** SMLM images showing the periodic pattern of ß4-spectrin along the AIS (d) or ß2-spectrin along the distal axon (e). **(f-g)** SMLM images showing the alternate periodic pattern of actin (gray) and ß4-spectrin along the AIS (f, orange) or ß2-spectrin along the distal axon (g, orange). **(h)** PREM view of an unroofed axon showing the regularly spaced braids (magenta, arrowheads). Scale bars 40 µm (a), 2 µm (b-g), 5, 2, 0.5 and 0.2 µm (h, left to right).

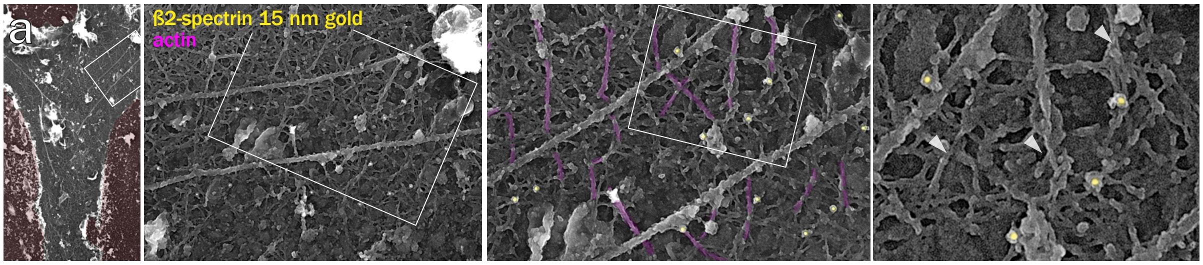

Supplementary Figure 2. Immunogold labeling of ß2-spectrin.
(a) PREM views of an unroofed axon immunogold-labeled (15 nm gold beads are pseudo-colored yellow) for ß2-spectrin between actin braids (magenta, arrowheads). Scale bars 2 µm, 1, 0.5, 0.2 µm (left to right).

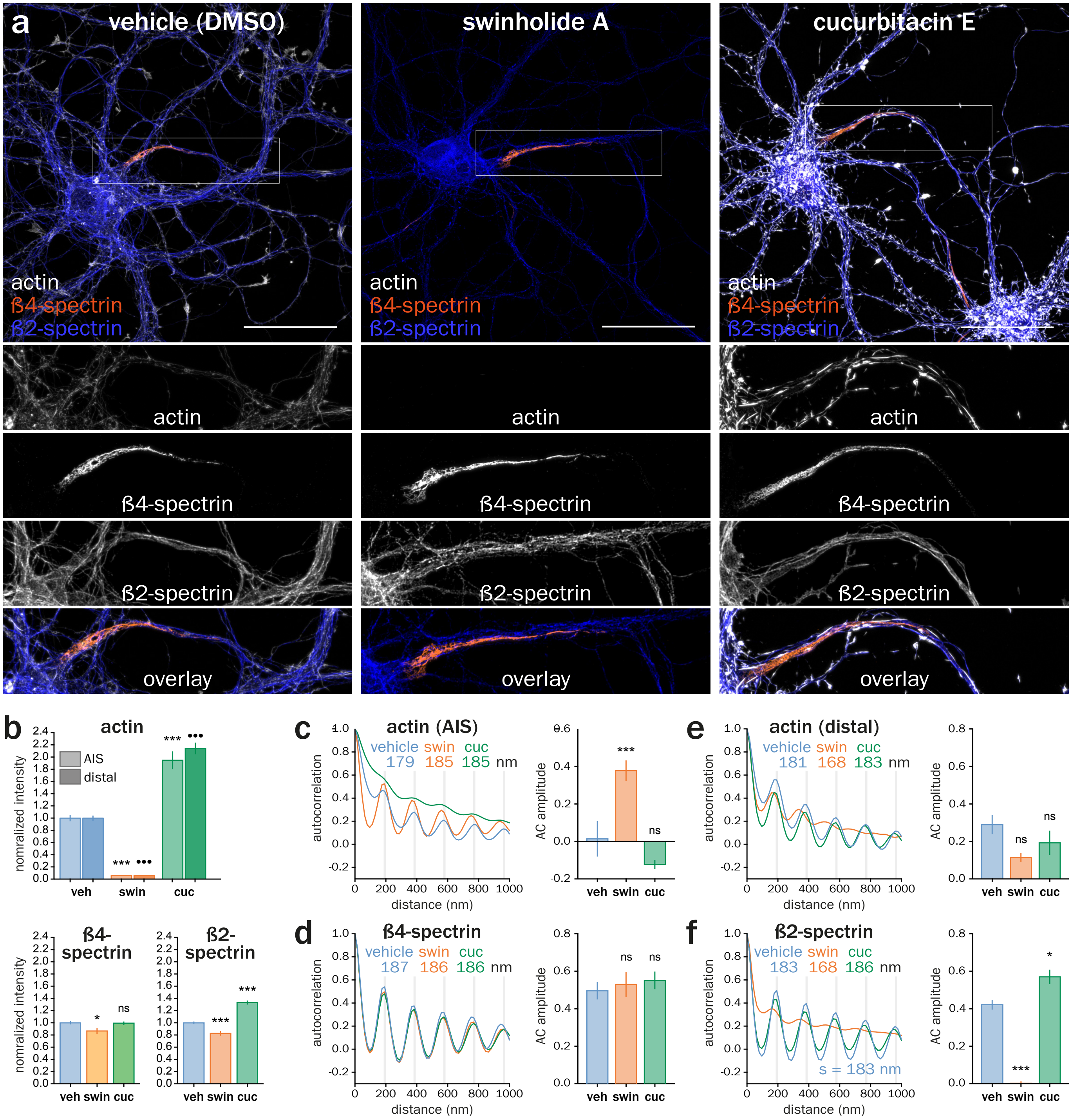

Supplementary Figure 3. Epifluorescence images and quantification of drugs effects on MPS components.
(a) Epifluorescence image of neurons treated for 3h with vehicle (DMSO 0.1%), swinholide A (100 nM) or cucurbitacin E (5 nM) and labeled for actin (gray), ß4-spectrin (orange) and ß2-spectrin (blue). **(b)** Top, normalized axonal labeling intensity for actin along the AIS (light) and distal axons (dark) measured on epifluorescence images of treated neurons. Bottom, same quantification for the ß4- and ß2-spectrin labeling (mean ± SEM, n=74-777 tracings from 3 to 7 independent experiments, ns p>0.05, * p<0.05, *** p<0001, ANOVA post-hoc test). **(c-f)** Left, autocorrelation curve of the labeling after drug treatment for actin along the AIS (c), ß4-spectrin (d), actin along distal axons (e), or ß2-spectrin (f). Spacing measured from the autocorrelation curve is indicated. Right, measurement of the corresponding autocorrelation amplitude for the different treatments (mean ± SEM, n=8-60 tracings from 2 to 6 independent experiments, ns p>0.05, * p<0.05, *** p<0001, ANOVA post-hoc test). Scale bar, 40 µm (a). Source data for graphs (b-f) are provided as a Source Data file.

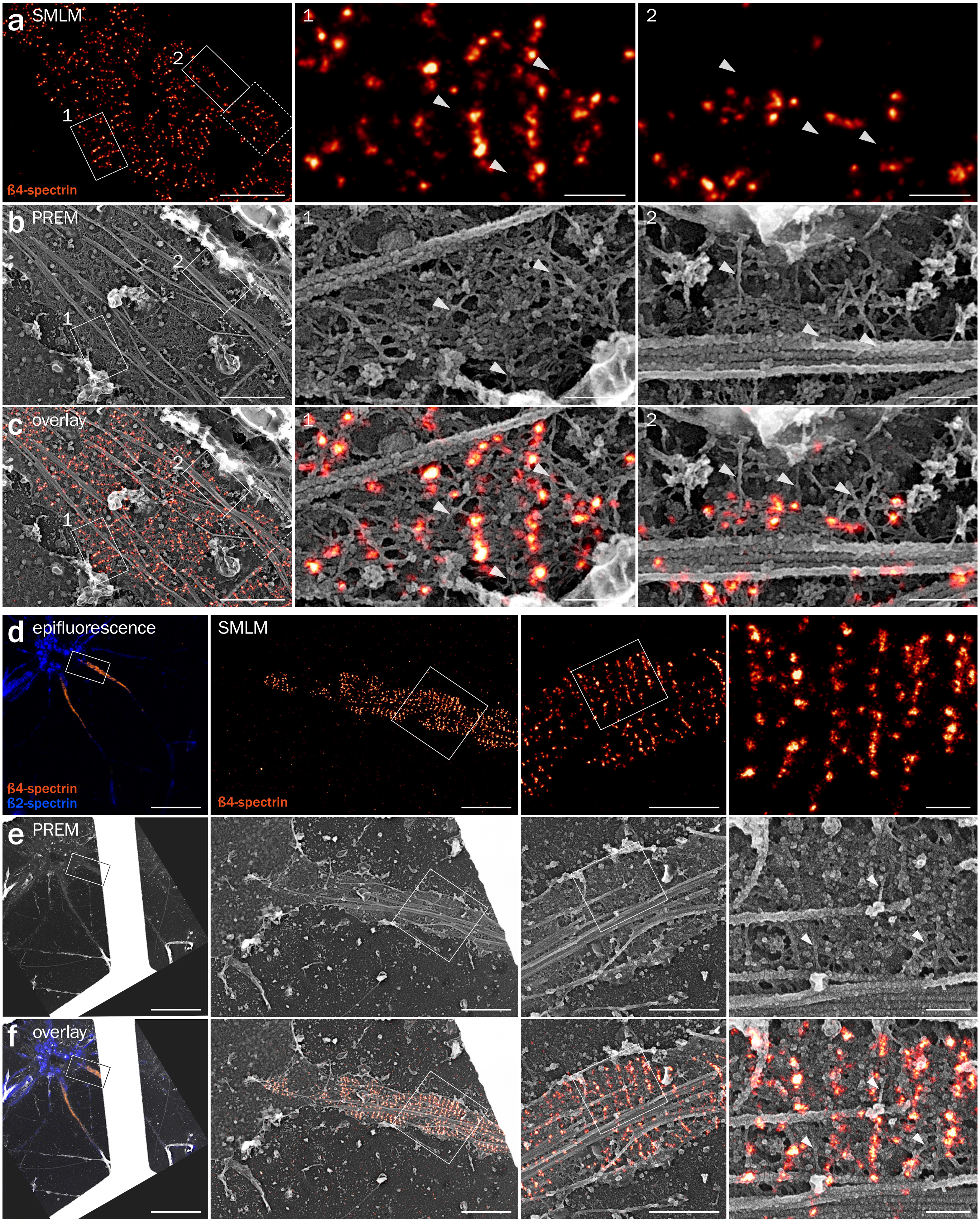

Supplementary Figure 4. Additional examples for correlative SMLM/PREM of ß4-spectrin
(a-c) Additional zooms from the SMLM/PREM image of ß4-spectrin along the axon shown in Fig. 5. Zoom shown in Fig. 5 is highlighted by a dotted box on the image on the left, and additional zooms 1-2 presented on the right are highlighted by solid boxes. SMLM image of ß4-spectrin labeling (a), corresponding PREM view (b) and overlay of the SMLM and PREM images (c). Scale bars 1, 0.2, 0.2 µm (from left to right). **(d)** Left, epifluorescence image of another unroofed neuron labeled for ß4-spectrin (orange) and ß2-spectrin (blue). Right, SMLM images of the unroofed axon labeled for ß4-spectrin. **(e)** Corresponding PREM views of the same unroofed neuron and axon (actin braids, arrowheads). **(f)** Overlay of the SMLM image and PREM views. Scale bars 20, 2, 1, 0.2 µm (from left to right).

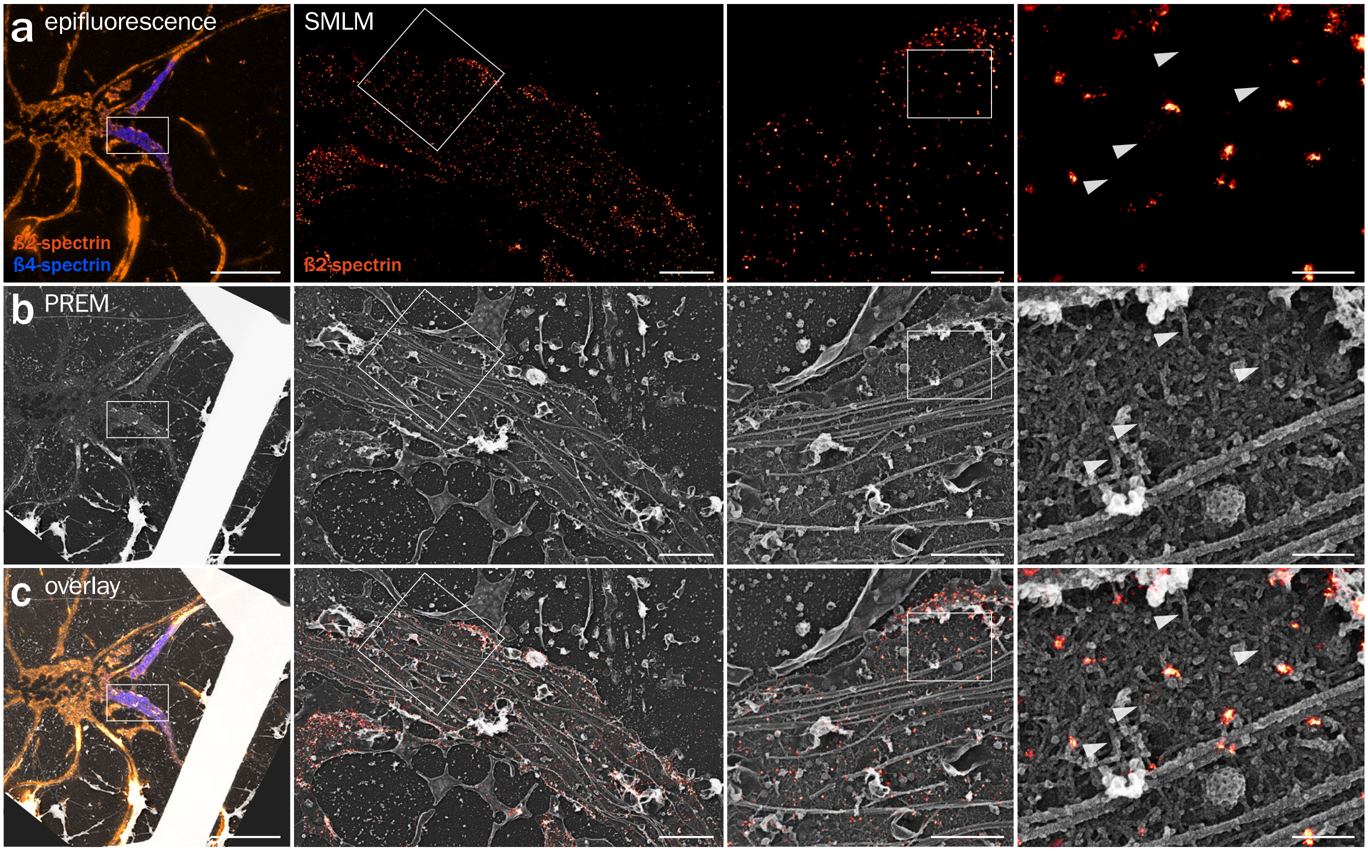

Supplementary Figure 5. Correlative SMLM/PREM of ß2-spectrin
(a) Left, epifluorescence image of an unroofed neuron labeled for ß2-spectrin (orange) and ß4-spectrin (blue). Right, SMLM images of the unroofed axon labeled for ß2-spectrin. **(b)** Corresponding PREM views of the same unroofed neuron and axon (actin braids, arrowheads). **(c)** Overlay of the SMLM image and PREM views. Scale bars 20, 2, 1, 0.2 µm (from left to right).

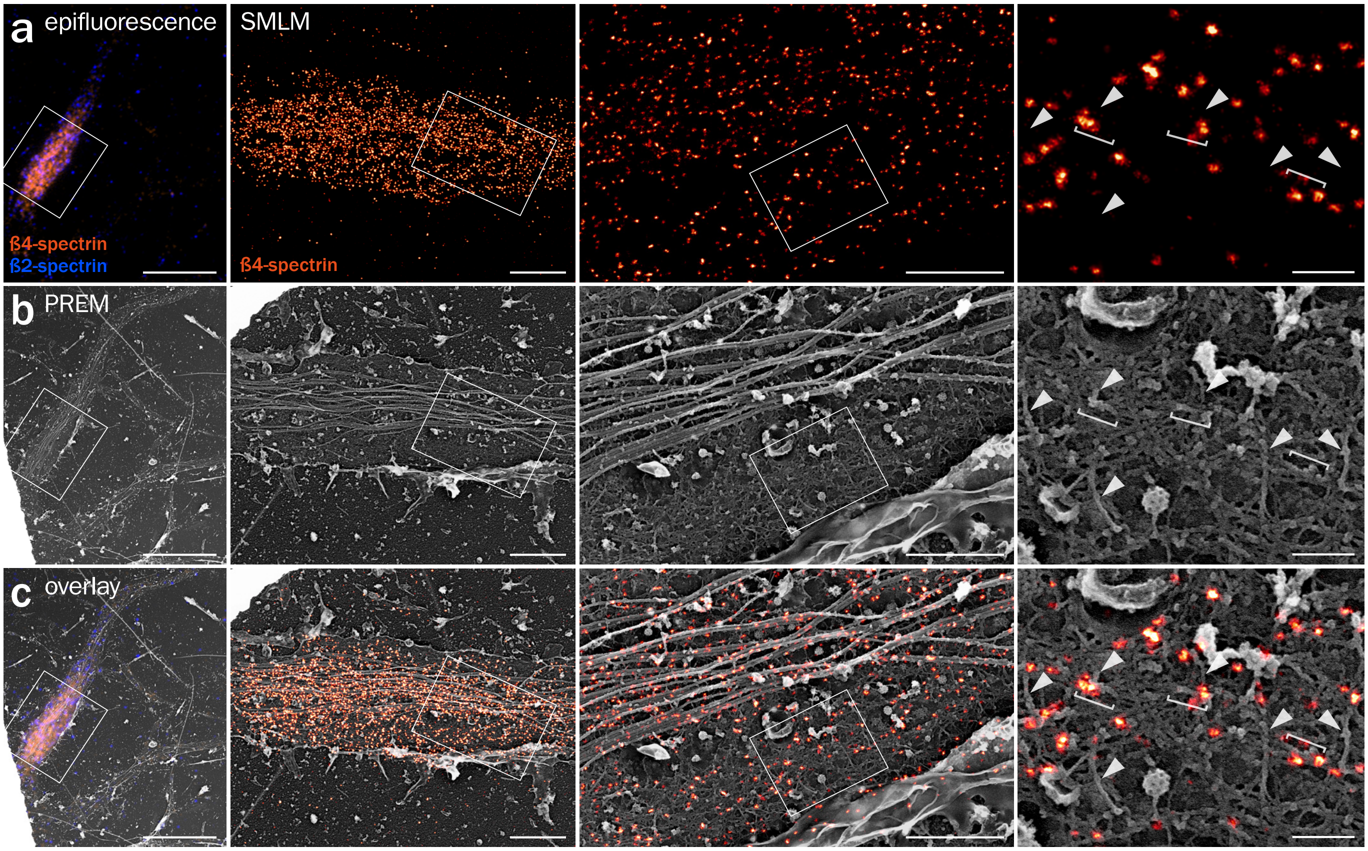

Supplementary Figure 6. Correlative SMLM/PREM of pMLC

(a) Left, epifluorescence image of an unroofed neuron labeled for pMLC (orange) and ankyrin G (blue). Right, SMLM images of the unroofed proximal axon labeled for pMLC. **(b)** Corresponding PREM views of the same unroofed neuron and axon. **(c)** Overlay of the SMLM image and PREM views showing pMLC apposed to actin breads (arrowheads). Scale bars 20, 2, 1, 0.2 µm (from left to right).

Supplementary Tables

		Intensity along axons from epifluorescence					Autocorrelation of axonal segments from SMLM						
		Normalized intensity		Number of data points			Spacing s (µm)		Amplitude		Number of data points		
Labeling	*Treatment*	*Mean*	*SEM*	*Tracings*	*Images*	*Indep. exp*	*Fit value*	*Error*	*Mean*	*SEM*	*Tracings*	*Images*	*Indep. exp*
actin (AIS)	vehicle	1,000	0,051	77	34	5	0,179	0,007	0,014	0,095	18	14	4
	unroofed						0,187	0,002	0,302	0,043	42	32	6
	swin	0,061	0,006	46	30	3	0,185	0,003	0,378	0,054	20	9	3
	cuc	1,948	0,146	65	24	3	0,142	0,287	-0,123	0,023	8	9	3
actin (distal)	ctrl	1,000	0,042	451	34	5	0,181	0,003	0,289	0,051	52	13	4
	swin	0,061	0,006	177	30	3	0,168	0,012	0,115	0,023	15	9	3
	cuc	2,142	0,095	283	24	3	0,183	0,004	0,192	0,064	27	9	3
ß4-spectrin	ctrl	1,000	0,026	107	56	6	0,187	0,002	0,497	0,047	43	14	3
	unroofed						0,191	0,002	0,527	0,070	16	9	2
	swin	0,867	0,047	74	52	4	0,186	0,002	0,530	0,067	25	12	2
	cuc	0,993	0,029	105	46	5	0,186	0,002	0,552	0,047	33	14	3
ß2-spectrin	ctrl	1,000	0,019	777	66	7	0,184	0,001	0,422	0,026	60	18	4
	unroofed						0,185	0,003	0,367	0,041	30	15	2
	swin	0,677	0,027	450	62	5	0,163	0,017	0,003	0,010	29	15	3
	cuc	1,285	0,031	502	56	5	0,188	0,001	0,570	0,038	31	11	2

Supplementary Table 1. Data statistics summary for fluorescence microscopy quantifications

		Number of data points	
Experiment	*Treatment*	*Images*	*Experiments*
Morphology	control	160	5
Immunogold	phalloidin	26	1
	ß4-spectrin	70	5
	ß2-spectrin	72	4
	α2/ß2/ß4-spectrin	34	1
	pMLC	44	3
	ankG	36	3
Actin labeling	myosin S1	51	2
Perturbation	swinholide A	30	2
	cucurbitacin E	20	2
Correlative	phalloidin	21	4
	ß4-spectrin	5	2
	ß2-spectrin	4	1
	pMLC	9	2
	ankG	7	2

Supplementary Table 2. Summary of image numbers for electron microscopy

		Analysis from PREM views				
		Value (nm)		Number of data points		
Measurement	*Treatment*	*Mean*	*SEM*	*Filaments*	*Images*	*Exp*
filaments spacing	control	183,8	4,5	50	10	5
filaments length	control	688,6	41,2	45	6	5
	myosin S1	1130,0	44,5	76	13	2
filaments thickness	braid	18,5	0,2	90	31	8
	split braid	10,2	0,3	52	18	8
	dendrite	9,9	0,2	60	5	3
	microtubule	31,0	0,7	33	6	5

Supplementary Table 3. Data statistics summary for electron microscopy quantifications

Captions for Supplementary Movies 1 to 7

Supplementary Movie 1. Tomogram corresponding to Fig. 1j.

PREM tomogram of an unroofed axon. Actin braids appear in magenta during the movie, then the spectrin mesh in yellow, and microtubules in blue.

Supplementary Movie 2. Tomogram corresponding to Fig. 1k.

PREM tomogram of an unroofed axon. Actin braids appear in magenta during the movie, then the spectrin mesh in yellow, and microtubules in blue.

Supplementary Movie 3. Tomogram corresponding to Supplementary Figure 1h.

PREM tomogram of an unroofed axon. Actin braids appear in magenta during the movie, then the spectrin mesh in yellow, and microtubules in blue.

Supplementary Movie 4. Tomogram corresponding to Fig. 2g.

PREM tomogram of an unroofed axon. Actin braids appear in magenta during the movie, then the spectrin mesh in yellow, and microtubules in blue.

Supplementary Movie 5. Correlative PREM/SMLM for actin corresponding to Fig 5a-c.

The unroofed neuron is shown with successive epifluorescence image (ß2-spectrin in green, ß4-spectrin in red, actin in blue), low-magnification PREM image (grid appears white), SMLM image (actin in orange) and high-magnification PREM image of the proximal axon. The high-magnification PREM image of the axon is then superimposed with the SMLM image (actin in orange).

Supplementary Movie 6. Correlative PREM/SMLM for ß4-spectrin corresponding to Supplementary Figure 4d-f.

The high-magnification PREM image of the axon is superimposed with the SMLM image (ß4-spectrin in orange).

Supplementary Movie 7. Correlative PREM/SMLM for ß2-spectrin corresponding to Supplementary Figure 5a-c.

The high-magnification PREM image of the axon is superimposed with the SMLM image (ß2-spectrin in orange).
