# Supplementary material for: Ultrastructure of the axonal periodic scaffold reveals a braid-like organization of actin rings
Source: Nat Commun. 2019 Dec 20;10:5803. doi: 10.1038/s41467-019-13835-6 (PMC6925202; doi:10.1038/s41467-019-13835-6)
Supplement: Supplementary file 9 — Reporting Summary [file 41467_2019_13835_MOESM9_ESM.pdf]

nature research

Corresponding author(s): Christophe Leterrier, Stéphane Vassilopoulos

Last updated by author(s): Jun 3, 2019

Reporting Summary

Nature Research wishes to improve the reproducibility of the work that we publish. This form provides structure for consistency and transparency in reporting. For further information on Nature Research policies, see Authors & Referees and the Editorial Policy Checklist.

Statistics

For all statistical analyses, confirm that the following items are present in the figure legend, table legend, main text, or Methods section.

n/a	Confirmed	
☐	☒	The exact sample size (*n*) for each experimental group/condition, given as a discrete number and unit of measurement
☐	☒	A statement on whether measurements were taken from distinct samples or whether the same sample was measured repeatedly
☐	☒	The statistical test(s) used AND whether they are one- or two-sided<br>*Only common tests should be described solely by name; describe more complex techniques in the Methods section.*
☒	☐	A description of all covariates tested
☒	☐	A description of any assumptions or corrections, such as tests of normality and adjustment for multiple comparisons
☒	☐	A full description of the statistical parameters including central tendency (e.g. means) or other basic estimates (e.g. regression coefficient) AND variation (e.g. standard deviation) or associated estimates of uncertainty (e.g. confidence intervals)
☒	☐	For null hypothesis testing, the test statistic (e.g. *F*, *t*, *r*) with confidence intervals, effect sizes, degrees of freedom and *P* value noted<br>*Give P values as exact values whenever suitable.*
☒	☐	For Bayesian analysis, information on the choice of priors and Markov chain Monte Carlo settings
☒	☐	For hierarchical and complex designs, identification of the appropriate level for tests and full reporting of outcomes
☒	☐	Estimates of effect sizes (e.g. Cohen's *d*, Pearson's *r*), indicating how they were calculated

Our web collection on statistics for biologists contains articles on many of the points above.

Software and code

Policy information about availability of computer code

Data collection	Sotware used (commercial): Nikon Elements, Zeiss Zen
Data analysis	Software used: Nikon N-STORM, Fiji/ImageJ. Custom Fiji/ImageJ code for data analysis is available at https://github.com/cleterrier?tab=repositories.

For manuscripts utilizing custom algorithms or software that are central to the research but not yet described in published literature, software must be made available to editors/reviewers. We strongly encourage code deposition in a community repository (e.g. GitHub). See the Nature Research guidelines for submitting code & software for further information.

Data

Policy information about availability of data

All manuscripts must include a data availability statement. This statement should provide the following information, where applicable:

- Accession codes, unique identifiers, or web links for publicly available datasets
- A list of figures that have associated raw data
- A description of any restrictions on data availability

Raw data from the data presented in the Figures of this manuscript are available from Figshare at the following link: https://figshare.com/s/7cf4262c6d922add64ac

Field-specific reporting

Please select the one below that is the best fit for your research. If you are not sure, read the appropriate sections before making your selection.

☒ Life sciences ☐ Behavioural & social sciences ☐ Ecological, evolutionary & environmental sciences

For a reference copy of the document with all sections, see nature.com/documents/nr-reporting-summary-flat.pdf

Life sciences study design

All studies must disclose on these points even when the disclosure is negative.

Sample size	No sample-size calculations were performed. Number of replicates and individual data points are similar or above standards in the field.
Data exclusions	No data exclusion was performed
Replication	Fluorescence microscopy experiments were replicated independantly (different neuronal cultures) at least 3 times. Extensive observation of electron microscopy samples was performed to ensure representative results. See Tables S1 and S2 of the manuscript for more details.
Randomization	No randomization was performed.
Blinding	No blinding was performed but quantification was realized using semi-automatic tracing scripts that minimize user variability and potential bias.

Reporting for specific materials, systems and methods

We require information from authors about some types of materials, experimental systems and methods used in many studies. Here, indicate whether each material, system or method listed is relevant to your study. If you are not sure if a list item applies to your research, read the appropriate section before selecting a response.

Materials & experimental systems

n/a	Involved in the study	
☐	☒	Antibodies
☒	☐	Eukaryotic cell lines
☒	☐	Palaeontology
☐	☒	Animals and other organisms
☒	☐	Human research participants
☒	☐	Clinical data

Methods

n/a	Involved in the study	
☒	☐	ChIP-seq
☒	☐	Flow cytometry
☒	☐	MRI-based neuroimaging

Antibodies

Antibodies used	Rabbit polyclonal anti ßIV-spectrin antibody (against residues 2237-2256 of human ßIV-spectrin, 1:800 dilution for immunofluorescence IF, 1:20 for immunogold IG) was a gift from Matthew Rasband (Baylor College of Medicine, Austin, TX). Mouse monoclonal anti ßII-spectrin (against residues 2101-2189 of human ßII-spectrin, 1:100 for IF, 1:20 for IG) was from BD Biosciences (#612563). Chicken anti-map2 antibody was from abcam (#ab5392, 1:1000 for IF). Rabbit polyclonal anti-Alexa Fluor 488 antibody was from Thermo Fisher (A11094, 1:20 for IG). Mouse monoclonal antibodies anti ankyrin G (clone 106/65 and 106/36, 1:300 for IF) were from NeuroMab. Rabbit polyclonal anti 480-kDa ankG (residues 2735-2935 of rat 480-kDa ankG, 1:300 for IF, 1:100 for IG) was a gift from Vann Bennett (Duke University, Durham, NC). Rabbit anti phospho-Myosin Light Chain 2 Thr18/Ser19 (pMLC) was from Cell Signaling Technologies (#3674, 1:50 for IF, 1:20 for IG).
Validation	Each antibody has been previously used in previously published studies, including our lab and is extensively validated.

Animals and other organisms

Policy information about studies involving animals; ARRIVE guidelines recommended for reporting animal research

Laboratory animals	Neuronal culture were made from embryonic Wistar rats (both sexes).
Wild animals	No wild animals used.
Field-collected samples	No field-collected samples used.
Ethics oversight	The use of Wistar rats followed the guidelines established by the European Animal Care and Use Committee (86/609/CEE) and was approved by the local ethics committee (agreement D13-055-8).

Note that full information on the approval of the study protocol must also be provided in the manuscript.
