# Supplementary material for: Ultrastructure of the axonal periodic scaffold reveals a braid-like organization of actin rings
Source: Nat Commun. 2019 Dec 20;10:5803. doi: 10.1038/s41467-019-13835-6 (PMC6925202; doi:10.1038/s41467-019-13835-6)
Supplement: Supplementary file 10 — Description of Additional Supplementary Files [file 41467_2019_13835_MOESM10_ESM.docx]

Captions for Supplementary Movies 1 to 7

**Supplementary Movie 1. Tomogram corresponding to Fig. 1j.**

PREM tomogram of an unroofed axon. Actin braids appear in magenta during the movie, then the spectrin mesh in yellow, and microtubules in blue.

**Supplementary Movie 2. Tomogram corresponding to Fig. 1k.**

PREM tomogram of an unroofed axon. Actin braids appear in magenta during the movie, then the spectrin mesh in yellow, and microtubules in blue.

**Supplementary Movie 3. Tomogram corresponding to Supplementary Figure 1h.**

PREM tomogram of an unroofed axon. Actin braids appear in magenta during the movie, then the spectrin mesh in yellow,and microtubules in blue.

**Supplementary Movie 4. Tomogram corresponding to Fig. 2g.**

PREM tomogram of an unroofed axon. Actin braids appear in magenta during the movie, then the spectrin mesh in yellow, and microtubules in blue.

**Supplementary Movie 5. Correlative PREM/SMLM for actin corresponding to Fig 5a-c.**

The unroofed neuron is shown with successive epifluorescence image (ß2-spectrin in green, ß4-spectrin in red, actin in blue), low-magnification PREM image (grid appears white), SMLM

image (actin in orange) and high-magnification PREM image of the proximal axon. The high-magnification PREM image of the axon is then superimposed with the SMLM image (actin in

orange).

**Supplementary Movie 6. Correlative PREM/SMLM for ß4-spectrin corresponding to Supplementary Figure 4d-f.**

The high-magnification PREM image of the axon is superimposed with the SMLM image (ß4-spectrin in orange).

**Supplementary Movie 7. Correlative PREM/SMLM for ß2-spectrin corresponding to Supplementary Figure 5a-c.**

The high-magnification PREM image of the axon is superimposed with the SMLM image (ß2-spectrin in orange).
